# The Effect of eHMI Malfunctions on Younger and Elderly Pedestrians’ Trust and Acceptance of Automated Vehicle Communication Signals

**DOI:** 10.3389/fpsyg.2022.866475

**Published:** 2022-05-03

**Authors:** Ann-Christin Hensch, Isabel Kreißig, Matthias Beggiato, Josef F. Krems

**Affiliations:** Cognitive and Engineering Psychology, Department of Psychology, Chemnitz University of Technology, Chemnitz, Germany

**Keywords:** automated vehicles, communication cues, external human–machine interface, system malfunctions, trust, acceptance, vulnerable road users, elderly pedestrians

## Abstract

To ensure traffic flow and road safety in automated driving, external human–machine interfaces (eHMIs) could prospectively support the interaction between automated vehicles (AVs; SAE Level 3 or higher) and pedestrians if implicit communication is insufficient. Particularly elderly pedestrians (≥65 years) who are notably vulnerable in terms of traffic safety might benefit of the advantages of additional signals provided by eHMIs. Previous research showed that eHMIs were assessed as useful means of communication in AVs and were preferred over exclusively implicit communication signals. However, the attitudes of elderly users regarding technology usage and acceptance are ambiguous (i.e., less intention to use technology vs. a tendency toward overreliance on technology compared to younger users). Considering potential eHMI malfunctions, an appropriate level of trust in eHMIs is required to ensure traffic safety. So far, little research respected the impact of multiple eHMI malfunctions on participants’ assessment of the system. Moreover, age effects were rarely investigated in eHMIs. In the current monitor-based study, *N* = 36 participants (19 younger, 17 elderly) repeatedly assessed an eHMI: During an initial measurement, when encountering a valid system and after experiencing eHMI malfunctions. Participants indicated their trust and acceptance in the eHMI, feeling of safety during the interaction and vigilance toward the eHMI. The results showed a positive effect of interacting with a valid system that acted consistently to the vehicle’s movements compared to an initial assessment of the system. After experiencing eHMI malfunctions, participants’ assessment of the system declined significantly. Moreover, elderly participants assessed the eHMI more positive across all conditions than younger participants did. The findings imply that participants considered the vehicle’s movements as implicit communication cues in addition to the provided eHMI signals during the encounters. To support traffic safety and smooth interactions, eHMI signals are required to be in line with vehicle’s movements as implicit communication cues. Moreover, the results underline the importance of calibrating an appropriate level of trust in eHMI signals. An adequate understanding of eHMI signals needs to be developed. Thereby, the requirements of different user groups should be specifically considered.

## Introduction

Pedestrians are the most vulnerable road user group when it comes to traffic accidents due to the high number of 20% of all road fatalities (European Commission, 2020). Since they are over-represented regarding severe injuries in case of accidents, elderly pedestrians (≥65 years) are particularly vulnerable in terms of traffic safety (European Commission, 2021). Therefore, this user group should be specifically considered when it comes to road safety. Automated vehicles (AVs, SAE Level 3 or higher) provide the potentials of increased road safety, traffic efficiency, and enhanced driving comfort (SAE, 2018). However, to benefit from increased automated driving functions, AVs need to provide safe and smooth interactions with manual traffic participants in- and outside the vehicle and need to be accepted (Habibovic et al., 2018). Thus, AVs’ interaction capabilities need to be transparent and predictable to prevent from breakdowns, provide a common ground of interactions, and thus intuitive and safe encounters with other road users (Clark and Brennan, 1991; Endsley, 1995). Therefore, established interaction capabilities of manual traffic participants should be considered to be prospectively implemented in AVs (Portouli et al., 2014).

Since traffic is a social system, the different participants use various information of the driving scene to anticipate and coordinate prospective movements (Wilde, 1976). A coordination of actions is particularly required in shared spaces, such as parking areas, that are characterized by a high number of potentially ambiguous encounters due to limited statutory regulations and a diversity of traffic participants, such as pedestrians and vehicles, that need to interact (Hamilton-Baillie, 2008). To resolve ambiguities and support traffic safety, the communication between different traffic participants is required. Thereby, road users apply implicit (e.g., trajectory) and explicit (e.g., turn indicator) signals to communicate (Dey and Terken, 2017; for an overview of pedestrian-driver interaction see Rasouli and Tsotsos, 2019). In AVs, interactions between drivers and surrounding traffic participants will prospectively change since the driver might potentially be engaged in other tasks than driving and will no longer be available as an interaction partner. Thus, established communication cues between drivers and pedestrians, such as eye contact, need to be substituted in AVs (Lundgren et al., 2017). External human–machine interfaces (eHMIs) might compensate for a potentially missing interaction between drivers and surrounding traffic participants (Schieben et al., 2019) and offer the potential to support interactions in AVs if implicit communication is insufficient (Ackermann et al., 2019).

The current study aimed at investigating the development of participants’ assessment of an eHMI as potential means of communication in AVs during repeated measures. Thereby, the influence of system experience, valid and invalid eHMI functions, and the effect of participants’ age on the system assessment was investigated.

### External Human–Machine Interfaces in Automated Vehicles

As potential communication signals in automated driving, eHMIs could provide additional information about the AVs’ state and thus supply feedback to other traffic participants and could prevent confusion of surrounding road users. Moreover, eHMIs have the potential to announce prospective driving maneuvers of AVs and support the anticipation of the prospective development of the traffic scenario. Therefore, eHMIs are assumed to support pedestrians’ situational awareness of the traffic scenario and could, in turn, enhance traffic safety (Endsley, 1995; Krems and Baumann, 2009; Habibovic et al., 2018). However, pedestrians need to consider the eHMI signals as a source of information to benefit of the additional information. Previous research could show that eHMIs as means of communication in AVs generally supported the interaction with surrounding traffic participants (for an overview see Rouchitsas and Alm, 2019), especially in shared space settings comprising a high number of ambiguous encounters between diverse traffic participants (Merat et al., 2018). In detail, participants indicated higher trust ratings (Faas et al., 2020), higher acceptance ratings (Schindler et al., 2020), and higher feeling of safety (Böckle et al., 2017; de Clercq et al., 2019) during encounters including eHMI signals compared to baseline conditions that exclusively comprised implicit communication signals, such as the vehicles’ movement (i.e., dynamic HMI; Bengler et al., 2020). Since trust and acceptance display essential factors for a system’s usage and the users’ reliance (Lee and See, 2004; Ghazizadeh et al., 2012), these concepts need to be further considered for eHMIs as means of communication in AVs.

### Trust in Automation and Influencing Factors

Trust in automation is an essential determinant for system usage and can be described as “the attitude that an agent will help achieve an individual’s goals in a situation characterized by uncertainty and vulnerability” (Lee and See, 2004, p. 51). To maintain safe interactions but also apply the benefits of automated systems, an *appropriate* level of trust in the automation, that matches the capabilities of the system, is required. An *inappropriate* level of trust, on the other hand, could either lead to distrust or overtrust in the system. Distrust describes an insufficient level of trust in a system, leading to non-usage and, in turn, a loss of the advantages of the technical system (Lee and See, 2004). In the context of eHMIs, distrust in the system would lead to pedestrians’ reduced willingness to use the provided information by eHMI signals (Faas et al., 2021). Whereas, overtrust would result if the users’ trust exceeds the system’s capabilities. The users’ overtrust in a system, as an attitude, leads to overreliance in the system’s capabilities as a behavioral aspect (Lee and See, 2004). With regard to eHMIs in AVs, overtrust implies an overreliance in the eHMI signals that could lead to insufficient considerations of implicit communication signals that are provided by the vehicle’s driving behavior (Faas et al., 2021). Hence, overtrust should be respected as an essential safety issue in eHMIs (Tabone et al., 2021). Considering trust calibration and influencing factors, Hoff and Bashir (2015) proposed a theoretical framework that considers three layers of trust. According to the framework, a person’s *dispositional trust* is a relatively stable trait over time and reflects the general tendency for trust in automation, which, for instance, is influenced by the users’ age. In addition, dynamic factors reflected in *situational trust* and *learned trust* are also reported to influence users’ trust in a system. In particular, experience with the system and its performance influence the users’ learned trust in a system. To facilitate an appropriate usage of eHMI signals if applied in AVs, an adequate trust calibration in eHMI signals and potentially influencing factors need to be further considered. As an influencing factor on the users’ learned trust (Hoff and Bashir, 2015), experience with a system was shown to support the development of the users’ trust in the automated system (Muir and Moray, 1996). The positive influence of system experience on users’ trust has been also shown for the technology of eHMIs. Faas et al. (2020) investigated the development of users’ trust in eHMIs in three sessions of encounters with the system over a period of three weeks. The authors reported a constant increase of users’ trust when gaining experience with the investigated eHMI (Faas et al., 2020).

Besides experience with the system, its performance and reliability were also shown to influence the users’ trust in a system. More specifically, system failures were shown to decrease the users’ trust in the automation (Lee and Moray, 1992). Considering eHMIs, potential malfunctions cannot be excluded if the systems are applied as means of communication in AVs (Holländer et al., 2019). In the context of this study, eHMI malfunctions imply a mismatch between vehicles’ movements as implicit communication cues and eHMI signals. With regard to traffic safety, pedestrians need to be aware of potential malfunctions of eHMIs and are required to react appropriately in such potentially hazardous situations. For instance, pedestrians need to consider vehicles’ implicit communication cues (e.g., trajectory) over the eHMI signals in such cases (Kaleefathullah et al., 2020). Thus, to maintain traffic safety but also apply the benefits of AVs and potential eHMI signals, an *appropriate* level of trust in eHMI signals, that matches the capabilities of the system, is required if eHMIs are applied in AVs (Lee and See, 2004). Previous studies that investigated eHMI malfunctions, realized the malfunctions by contradicting information of the provided eHMI signals and the vehicles’ driving behavior as implicit communication signals. First research results indicated a decline of participants’ trust after encountering invalid eHMI functions in street crossing scenarios (Kaleefathullah et al., 2020; Faas et al., 2021). Faas et al. (2021) reported that participants’ trust declined temporarily after experiencing a single eHMI malfunction. Therefore, the authors concluded that participants’ trust formation in eHMIs can be seen as a dynamic process that is based on previous experience during encounters with the system (Faas et al., 2021). In order to prevent potentially safety critical situations and the users’ overtrust, the effect of multiple eHMI malfunctions was investigated in the current study in a shared space setting, comprising ambiguous encounters between the involved traffic participants.

### Effects of Invalid System Functions on Acceptance, Feeling of Safety, and Vigilance Toward the Automated System

A further essential predictor for system usage, which is strongly related to trust in automation, is the acceptance of an automated system (Ghazizadeh et al., 2012; Nordhoff et al., 2019). In the current study, the acceptance of a system will be defined as the users’ “direct attitude towards a system” according to Van Der Laan et al. (1997, p. 2). In the context of eHMIs as means of communication in AVs, the signals need to be accepted by pedestrians to benefit from the provided information of the system. Generally, previous research reported a benefit of eHMI signals for pedestrians when encountering AVs (Rouchitsas and Alm, 2019). With regard to traffic safety and the intention to use the information provided by an eHMI, pedestrians’ acceptance of the system also needs to be investigated in case of eHMI malfunctions, which might be unexpected for the pedestrians (Venkatesh et al. 2003). Beggiato and Krems (2013) investigated the effect of omitted system failures of an adaptive cruise control, as a form of driving assistance systems, on the users’ acceptance during multiple driving simulator sessions. The authors reported a sharp decline of the users’ acceptance when experiencing omitted system failures of the investigated driving assistance system (Beggiato and Krems, 2013). Since previous research reported a decline of participants’ acceptance due to invalid system’s functions, the influence of eHMI malfunctions on participants’ acceptance should be also investigated.

Besides potentially impairing the users’ acceptance, invalid system functions might also influence additional aspects of the interaction with eHMIs. For instance, Holländer et al. (2019) reported that even a single eHMI malfunction reduced the participants’ perceived safety during encounters with a vehicle in a simulated street crossing scenario. Moreover, due to the contradicting information between the eHMI signal and the interaction vehicle’s driving behavior, participants’ confidence regarding the vehicle’s prospective driving behavior declined significantly (Holländer et al., 2019). In addition, the supervisors’ vigilance toward a system represents an essential component to detect system failures and thus support safe interactions with automated systems, such as AVs. However, vigilance toward a system demands additional mental workload for monitoring the automated system (Warm et al., 2008). In the context of automated driving, vigilance was described as “state or degree of readiness to detect and to react to small changes in the environment that appear in random intervals” (Körber et al., 2015, p. 71). To gain more insight on the effects of eHMI malfunctions, the current study investigated multiple malfunctions and repeatedly examined participants’ assessment of the system regarding, trust, acceptance, perceived safety during the interaction and vigilance toward the system.

### Age Effects in eHMI Assessment and Traffic Safety

Signals provided by eHMIs and potential system malfunctions might be assessed differently among various user groups. Since elderly pedestrians (≥65 years) are over-represented regarding severe injuries in case of accidents, this user group is particularly vulnerable in terms of traffic safety (European Commission, 2021). Therefore, elderly pedestrians might particularly benefit of increased road safety as an advantage of AVs. Since eHMI signals are assumed to support pedestrians’ situational awareness by providing additional information of the traffic scene (Endsley, 1995; Habibovic et al., 2018), the signals might compensate for age-related declines, such as cognitive and sensory abilities as well as psycho-motoric functions of elderly (for an overview see Dunbar et al., 2004; Polders et al., 2015). According to the trust framework by Hoff and Bashir (2015), an influencing aspect of dispositional trust is reflected in the users’ age. However, there are ambiguous findings regarding elderly users’ attitudes toward technology. On the one hand, elderly users’ reported lower actual usage rates, less interest to use technology (Czaja et al., 2006), and reduced comfort when interacting with technology compared to younger users (Czaja and Sharit, 1998). In contrast, it was also reported that elderly users were more likely to trust automated systems (for an overview see Schaefer et al., 2016) and indicated a more positive attitude toward automated systems (e.g., Rödel et al., 2014; Hartwich et al., 2019). When investigating light-based eHMI signals in a field study, elderly participants indicated higher usefulness ratings (i.e., acceptance ratings) of the investigated signals than younger participants. The results might be constituted in elderly participants’ awareness that eHMI signals could provide additional information of driving scenes and might therefore compensate for age-related impairments, which could enhance traffic safety (Hensch et al., 2019b).

With regard to invalid functions of automated systems, Ho et al. (2005) compared younger and elderly participants’ trust and reliance on an automated decision aid. It was shown that elderly users were less sensitive in case of system failures and showed a tendency of overreliance on the system. Moreover, elderly users adjusted their trust in case of invalid functions of the automation aids less than younger users (Ho et al., 2005). Due to several age-related impairments (Dunbar et al., 2004; Polders et al., 2015) and the ambiguous relation between elderly and their attitude toward technology (Czaja et al., 2006; Schaefer et al., 2016), this specific user group needs to be particularly considered when it comes to eHMIs and potential malfunctions including possible safety issues. Currently, age-related differences in eHMI assessment are rarely investigated (as exceptions see Othersen et al., 2018; Hensch et al., 2019b). For this reason, the current study specifically investigated the effect of eHMI malfunctions on elderly participants (≥65 years) assessment of the system.

### Research Questions and Hypothesis

Since previous research reported a benefit of eHMIs for the communication in AVs, these signals seem a promising approach to support prospective interactions between AVs and surrounding traffic participants (Rouchitsas and Alm, 2019). Particularly in shared spaces with ambiguous encounters and diverse traffic participants interacting (Hamilton-Baillie, 2008), eHMIs might potentially support the communication and enhance traffic safety (Habibovic et al. 2018). However, with regard to safety aspects, pedestrians need to be aware of potential eHMI malfunctions (Holländer et al., 2019). Therefore, the current study investigated the effect of eHMI experience and repeated eHMI malfunctions in a shared space scenario. The influence on participants’ trust, acceptance, feeling of safety, and vigilance toward the eHMI was examined considering an elderly and a younger age group. Thereby, valid eHMI functions (i.e., match between vehicle’s movements and the eHMI signals) and invalid eHMI functions (i.e., mismatch between vehicle’s movements and the eHMI signals resulting in system malfunctions) were manipulated across three points of measurement:

‒ (t0) initial measurement (encountering the eHMI signals without being introduced in the study’s scenario of the parking lot as a shared space),
‒ (t1) measurement with system experience comprising exclusively valid system functions,
‒ (t2) measurement with system experience comprising valid and invalid system functions.

Thus, the first research question (RQ) addressed within the study is: How does participants’ trust in eHMIs develop across the points of measurement (RQ1)? Based on previous findings that reported an increase of users’ trust when gaining experience with an eHMI (Faas et al., 2020) and a decline of trust after experiencing system malfunctions (Kaleefathullah et al., 2020), it is assumed that: (H1a) Participants’ trust increases after experiencing exclusively valid system functions compared to the initial measurement (t0 < t1); (H1b) Participants’ trust in eHMIs decreases after experiencing multiple system malfunctions compared to exclusively valid system functions (t1 > t2).

The second RQ considers participants’ acceptance of the system: How does participants’ acceptance of eHMIs develop across the points of measurement (RQ2)? Based on findings by Beggiato and Krems (2013) who reported a decrease of the users’ acceptance after experiencing system failures that were not introduced beforehand, it is assumed that (H2): Participants’ acceptance in eHMIs decreases after experiencing system malfunctions compared to exclusively valid system functions (t1 > t2).

Furthermore, the current study examined the development of participants’ reported feeling of safety and vigilance toward the system as an indicator for participants’ awareness of potential eHMI malfunctions after interacting with a valid (t1) and an invalid system (t2). Therefore, the following RQs are investigated: How is participants’ feeling of safety affected by eHMI malfunctions (RQ3)? How is participants’ vigilance toward the eHMI affected by system malfunctions (RQ4)?

Based on the specific relevance due to the high vulnerability of elderly pedestrians in case of accidents (European Commission, 2021) but also ambiguous findings regarding attitudes of technology acceptance and usage by elderly (Czaja et al. 2006; Schaefer et al. 2016), it is of specific importance to investigate this user group regarding the means of communication between AVs and pedestrians. Thus, younger (18–40 years) and elderly participants’ (≥65 years) assessment of an eHMI is examined and compared for the different stages of system experience. This leads to the following research question addressed within the study: How do the investigated age groups differ regarding the assessment of the eHMI as potential means of communication in AVs across the different points of measurement (RQ5)?

## Materials and Methods

### Research Design

The current study investigated the effects of eHMI malfunctions (i.e., a mismatch between implicit communication cues of the vehicle’s movements and eHMI signals) on participants’ assessment of the system. A 3 (points of measurements, within-subject factor) x 2 (age groups, between-subjects factor) mixed design, with repeated measures on the points of measurements, was applied. The participants repeatedly assessed the system during three points of measurements [initial measurement (t0); after experiencing valid eHMI functions (t1); and after experiencing valid and invalid eHMI functions (t2)]. To investigate age-related differences of the eHMI assessment, participants’ age groups (18–40 years vs. ≥65 years) were applied as a between-subjects variable. The participants indicated their trust in and acceptance of the eHMI (t0–t2) as well as their feeling of safety during the interaction and the vigilance toward the eHMI (t1 and t2) as dependent variables.

### Material

#### Video Material

**Figure 1:**
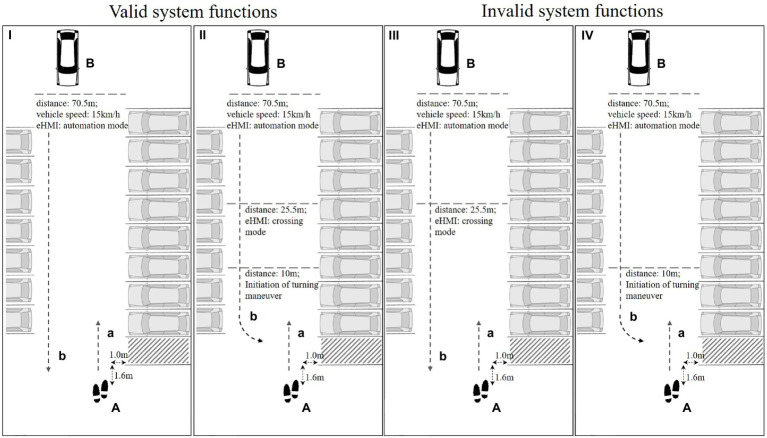
Schematic overview of the investigated scenario in the parking lot (top view). (A) The footprints represent the pedestrians’ perspective (i.e., the camera’s position), (a) displays the participants’ instructed trajectory for crossing the empty parking space (i.e., dashed area). (B) The vehicle indicates the approaching interaction vehicle including an augmented light-based eHMI in the windscreen, (b) displays the respective trajectory of the interaction vehicle that was recorded in the videos. The displayed distances of the interaction vehicle to video start, the transition of eHMI signals (when crossing mode was presented), and the initiation of the turning maneuver are displayed with respect to the camera’s position (i.e., the pedestrians’ perspective). (I and II) Valid system functions; (III and IV) invalid system functions.

The study applied real-world videos as study material displaying a straight encountering vehicle in a shared space setting. The videos were presented in a simulation environment that allowed for experimental control and standardized instructions. The environment was presented on a 28″ screen to the participants and was programmed in LabView (National Instruments, 2015). The videos were recorded on a parking area of Chemnitz University of Technology (Germany) by a GARMIN VIRB Ultra 30 (1920 × 1080 pixels, 100 fps). The position of the camera was set up to indicate a pedestrian’s perspective standing in front of an empty parking space that the participants were instructed intending to cross. To provide a realistic impression of the scenario, the camera was placed on a tripod at a height of 1.70 m in front of an empty parking space (Figure 1). The encountering interaction vehicle (BMW i3) approached with a speed of 15 km/h. A light-based eHMI in cyan color (*R* = 31/*G* = 237/*B* = 255) was augmented in the windscreen of the encountering vehicle with Adobe After Effects (Adobe Inc., 2020). To create valid and invalid eHMI functions, two augmented light-based eHMI signals and two different videos that displayed different trajectories of the approaching vehicle were applied (for an overview of the resulting conditions see Table 1; Figure 1).

**Table 1:** Overview of the valid and invalid eHMI functions resulting from the vehicle’s movements as implicit communication signals and the eHMI signals.

		Vehicle movement	
		Driving straight ahead	Left-turn maneuver
eHMI signal	Automation mode	Valid system function (F I; see also Figure 1 (I))	System malfunction (F IV; see also Figure 1 (IV))
	Crossing mode	System malfunction (F III; see also Figure 1 (III))	Valid system function (F II; see also Figure 1 (II))

**Figure 2:**
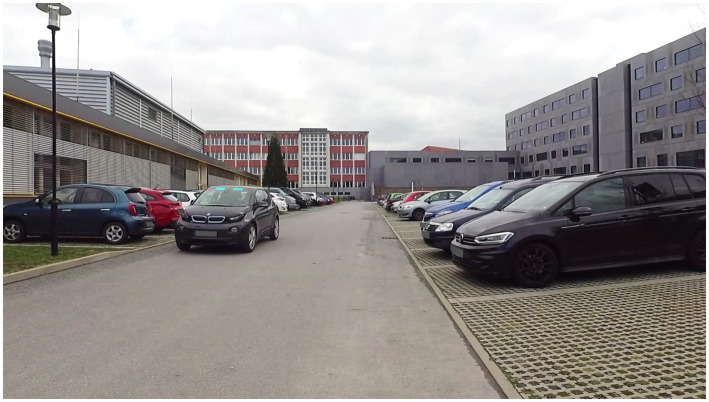
Screenshot of the applied video material displaying the encountering interaction vehicle driving straight ahead the parking lot with the augmented light-based eHMI (signal: automation mode) from the pedestrians’ perspective standing in front of an empty parking space the participants were instructed intending to cross.

**Figure 3:**
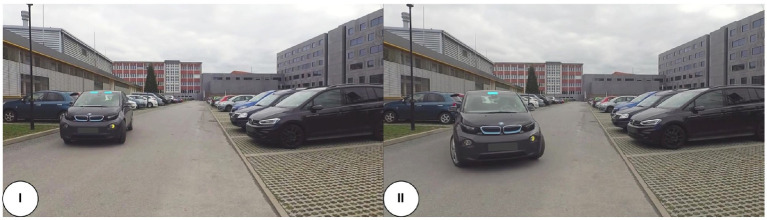
Screenshots of the applied video material displaying the interaction vehicle initiating a left-turn maneuver into the empty parking space in front of the participants with the augmented light-based eHMI (signal: crossing mode) and the activated turn indicator. (I) The interaction vehicle initiated a left-turn maneuver (including deceleration, changes in trajectory, and steering of tires); (II) the interaction vehicle further conducted left-turn maneuver, video stop.

With regard to the augmented light-based eHMI, a light bar in the windscreen of the vehicle displayed two abstract signals to the participants (Hensch et al., 2019a):

‒ Automation mode (screenshot of the signal see Figure 2): the automation mode displayed a steady light signal that intended to indicate that the vehicle was driving automated. In the respective conditions (Table 1), the automation mode was presented during the entire video [Figure 1 (I)].
‒ Crossing mode (screenshots of the signal see Figure 3): the crossing mode displayed a sweeping light signal that intended to indicate that the vehicle in automation mode would yield and the pedestrian could cross the empty parking space in front of the vehicle. In the respective conditions (Table 1), the automation mode was activated at the beginning of the trials and then switched to the crossing mode signal [Figure 1 (II)].

The moment of transition between automation mode and crossing mode was selected as a trade-off considering an unrealistically early presentation of the crossing mode and providing a sufficient display duration of the signal that participants could recognized the crossing mode signal. When the crossing mode was displayed by the eHMI, the turn signal of the interaction vehicle was activated simultaneously, to act in line with the road traffic regulations and to highlight the initiation of the upcoming left-turn maneuver into the empty parking space.

Both videos started displaying the interaction vehicle approaching to the camera’s position (i.e., the pedestrians’ position) and either:

‒ Driving straight ahead: the vehicle went with a constant speed straight ahead the parking lot and passed the pedestrians’ position without interfering the instructed hypothetical trajectory of the pedestrian (video duration: 18.95 s; Figure 1 (I); example screenshot of the maneuver see Figure 2) or
‒ Left-turn maneuver: the vehicle approached and initiated a left-turn maneuver into the empty parking space in front of the camera’s position (including changes in trajectory and deceleration), resulting in an overlap of the vehicle’s and the pedestrian’s hypothetical trajectories. This maneuver would have required the interaction vehicle to stop and give the pedestrian the priority of way to hypothetically cross the parking space in front of the vehicle [video duration: 18.10 s; Figure 1 (II); example screenshots of the maneuver see Figure 3].

Thus, the two light-based eHMI signals and the two movement conditions of the vehicle resulted in the following experimental conditions [for an overview see Figure 1 and Table 1]:

‒ (F I) Valid system function: eHMI displayed automation mode; vehicle went straight ahead the parking lot [Figure 1 (I)].
‒ (F II) Valid system function: eHMI displayed automation mode at the beginning of the video, transition to crossing mode and turn signal activated; vehicle initiated left-turn maneuver into empty parking space [i.e., dashed area; Figure 1 (II)].
‒ (F III) Invalid system function: eHMI displayed automation mode at the beginning of the video, transition to crossing mode and turn signal activated; vehicle went straight ahead the parking lot [Figure 1 (III)].
‒ (F IV) Invalid system function: eHMI displayed automation mode; vehicle initiated left-turn maneuver into empty parking space [i.e., dashed area; Figure 1 (IV)].

#### Questionnaires

All questionnaires were presented computer-based. Before the experimental blocks, a questionnaire was applied collecting socio-demographic information, such as participants’ specific age and gender. This questionnaire also contained standardized scales collecting participants’ affinity for technology interaction (ATI; Franke et al., 2019) and propensity to trust (Körber, 2019). The 9-item affinity for technology interaction scale according to Franke et al. (2019) was used to assess participants’ ATI. Participants indicated their agreement to the items on a 6-point Likert scale from [1] *“completely disagree”* to [6] *“completely agree”* that were aggregated to an overall score (Cronbach’s *α* = 0.86). Moreover, participants’ propensity to trust was collected with the trust in automation scale (Körber, 2019; subscale: propensity to trust). The participants stated their agreement to the three items on a 5-point Likert scale from [1] *“strongly disagree”* to [5] *“strongly agree.”* Afterward, the scores were averaged to an overall score (Cronbach’s *α* = 0.51, which however depicts a rather low reliability; Field, 2009).

To draw a valid picture of the development of participants’ assessment of the eHMI, trust, acceptance as well as feeling of safety during the interaction and participants’ vigilance toward the eHMI were repeatedly collected. For trust, the trust in automation scale according to Jian et al. (2000) was applied at t0 to t2, comprising 12 items, which were answered on a 7-point Likert scale ranging from [1] *“not at all”* to [7] *“absolutely.”* The items were afterward averaged, resulting in an overall trust score (Cronbach’s *α* = 0.86–0.96). Moreover, the Van der Laan acceptance scale (Van Der Laan et al., 1997) was applied during the initial measurement and after each experimental block (t0–t2) since participants’ acceptance of the eHMI was investigated during the study. The scale comprises two subscales: The subscale *usefulness*, which covers practical aspects of the system (5 items) and the subscale *satisfaction*, which describes comfort aspects when interacting with the system (4 items, Van Der Laan et al., 1997). Participants indicated their answers to the respective items on a five-point semantic differential (e.g., useful vs. useless) that was coded from [−2] to [+2] (*usefulness*: Cronbach’s *α* = 0.78–0.91; *satisfaction*: Cronbach’s *α* = 0.78–0.89). Moreover, participants’ feeling of safety during the interaction was collected with a single item measurement at t1 and t2 (“I felt safe when interacting with the vehicle”; adapted from Hensch et al., 2019a). The participants indicated their agreement on a 7-point Likert scale from [1] *“I completely disagree”* to [7] *“I completely agree*. In addition, the vigilance toward the eHMI was collected at t1 and t2 by a single item measurement (“I am vigilant towards the eHMI and its functions”; self-designed) on a scale ranging from [0] *“not at all”* to [100] *“totally.”*

### Procedure

At first, participants were welcomed and informed about the scope of the study. Moreover, informed consent was obtained. Afterward, participants completed an initial questionnaire comprising questions regarding socio-demographics as well as ATI and propensity to trust. Written instructions that contained information about AVs in general and the concept of eHMIs as potential means of communication in AVs were provided to standardize the given information. Additionally, the applied eHMI signals were presented and their general meaning was explained to the participants by pictures and short videos. The written explanations and pictures regarding the meaning of the applied eHMI signals were available during the entire study, so that participants could reassure regarding the signals’ meaning. There was no information about potential malfunctions of the eHMI provided to the participants. To ensure for participants’ comprehension of the concept of eHMIs, a control questions had to be answered. In case the control question was not answered correctly, participants received an additional explanation about the applied eHMI concept.

**Figure 4:**
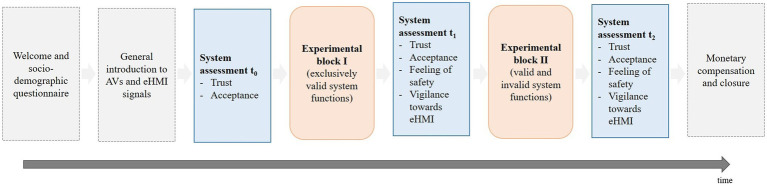
Procedure of the current study.

In a next step, all participants received a short repetitive explanation of the applied eHMI signals by videos and assessed the eHMI regarding trust and acceptance without any further instructions and without being introduced in the scenario at the parking lot (t0). Then, the scenario of the study in the parking lot was described and participants were instructed to take the perspective of a pedestrian intending to cross an empty parking space in front (Figure 1). The applied eHMI signals (i.e., automation mode and crossing mode) were explained with respect to the specific scenario in the parking lot. To prevent from fatigue, participants were instructed to indicate when they would no longer cross the empty parking space in front of the encountering vehicle by pressing the enter key. Moreover, a potential revision of this decision (i.e., crossing the empty parking space again) could have been also indicated by pressing the enter key again. To provide feedback to the participants regarding the decision, a green or red symbol in the simulation environment displayed the current state of the crossing decision (default setting: crossing the empty parking space, represented by a green symbol). To become familiarized with the eHMI signals in the parking area scenario and the instructed task, participants experienced six test trials including exclusively valid system functions. Afterward, participants experienced experimental block I comprising 18 randomized trials displaying the oncoming vehicle and the eHMI with exclusively valid system functions of the eHMI (nine trials of each valid system function, respectively; Table 1). Subsequently, participants evaluated the system regarding trust, acceptance, feeling of safety during the interaction and their vigilance toward the eHMI (t1). Again, to support the participants’ comprehension of the signals’ meanings, they received a reminder of the eHMI signals. Then, experimental block II with further 18 trials followed. Experimental block II comprised twelve valid (six trials of each valid system function, respectively; Table 1) and six invalid system functions (three trials of each type of malfunction, respectively; Table 1). The trails were presented in a balanced, determined order to control for influencing effects on the subsequent system assessment. Again, participants assessed the system afterward regarding trust, acceptance, feeling of safety, and vigilance toward the eHMI (t2). In the end, questions may had arisen were answered and all participants received a monetary compensation of 15€ for contributing to the study, which in sum lasted about one hour. See Figure 4 for an overview of the study’s procedure.

### Sample

Since one aim of the current study was to compare the eHMI assessment of different age groups, participants were divided into an elderly (≥65 years) and a younger group (18–40 years). In total, *N* = 37 participants contributed to the study. Due to answering the control question incorrect, one participant had to be excluded for further analysis. This resulted in a final sample of *n* = 36 participants (19 women, 17 men) across both age groups. In the group of younger participants (*n* = 19), *n* = 8 participants reported that no vision correction was required, whereas *n* = 11 participants reported corrected vision. Among the group of elderly participants (*n* = 17), all participants reported corrected vision. Further details of the sample and both experimental groups are provided in Table 2. To check for the age groups’ comparability and to control for other systematic group differences, the ATI scores and propensity to trust scores were compared between the groups. There was no difference for ATI between the age groups [*t*(34) = −0.02, *p* = 0.983, *d* = −0.01]. In addition, there was also no difference in propensity to trust between the two groups [*t*(34) = 0.02, *p* = 0.984, *d* = 0.01].

**Table 2:** Overview of the sample characteristics.

Age group	N	n_female_	n_male_	M_age_	SD_age_	Min_age_	Max_age_	M_ATI score_	SD_ATI score_	M_propensity to trust_	SD_propensity to trust_
Younger participants (18–40 years)	19	12	7	30.47	4.65	23	38	4.22	0.82	3.83	0.74
Elderly participants (≥65 years)	17	7	10	71.00	3.87	65	77	4.23	1.02	3.82	0.82

## Results

In the current study, mixed ANOVAs were applied. The assessment of the eHMI during the initial measurement of the system (t0), after experiencing valid system functions (t1), and after experiencing valid system functions and malfunctions (t2) served as within-subject factors. Participants’ age groups were applied as between-subjects factor (younger: 18–40 years vs. elderly: ≥65 years). Participants’ trust in and acceptance of the system, reported feeling of safety during the interaction and vigilance toward the eHMI served as dependent variables. The assumptions for parametric analysis (i.e., normal distribution, homogeneity of variances, and assumption of sphericity) were tested for each dependent variable and were given in most cases. In cases where the assumption of sphericity (Mauchly’s test) had been violated (*p* < 0.05), Greenhouse–Geisser corrected (Greenhouse–Geisser *Ɛ* ≤ 0.75) or Hyunh-Feldt corrected (Greenhouse–Geisser *Ɛ* > 0.75) *F*-values and degrees of freedom are reported. Extreme outliers were identified using boxplots (i.e., ≥three interquartile ranges over the third or under the first quartile). During the visual analysis, two outliers were identified in vigilance toward the eHMI and were therefore excluded for further analysis. An overview of the ANOVA results can be found in Table 3.

**Table 3:** Mixed ANOVA results displaying the main and interaction effects of the investigated factors points of measurement (within-subject factor) and participants’ age groups (between-subjects factor).

Measurement	Effect	df1, df2	*F*-value	*p*	*η^2^_p_*
Trust	**Point of measurementb**	**1.66, 57.91**	**23.78**	**<0.001**	**0.400**
	**Age group**	**1, 34**	**10.73**	**0.002**	**0.240**
	Point of measurement x age group^b^	1.69, 57.57	1.71	0.193	0.048
Acceptance: usefulness	**Point of measurementa**	**1.43, 50.12**	**11.26**	**<0.001**	**0.243**
	**Age group**	**1, 34**	**6.33**	**0.017**	**0.157**
	**Point of measurement x age groupa**	**1.49, 50.80**	**4.54**	**0.024**	**0.118**
Acceptance: satisfaction	**Point of measurementb**	**1.65, 57.60**	**8.69**	**0.001**	**0.199**
	Age group	1, 34	2.95	0.095	0.080
	**Point of measurement x age groupb**	**1.77, 60.03**	**3.36**	**0.047**	**0.090**
Feeling of safety	**Point of measurement**	**1, 35**	**49.68**	**<0.001**	**0.587**
	**Age group**	**1, 34**	**4.43**	**0.043**	**0.115**
	Point of measurement x age group	1, 34	0.43	0.518	0.012
Vigilance toward the eHMI	**Point of measurement**	**1, 33**	**4.89**	**0.034**	**0.129**
	Age group	1, 32	3.86	0.058	0.108
	Point of measurement x age group	1, 32	2.89	0.099	0.083

*Statistically significant results are highlighted in bold*.

a *Greenhouse-Geisser corrected degrees of freedom are reported*.

b *Hyunh-Feldt corrected degrees of freedom are reported. *N* = 36*.

### Trust in Automation

**Figure 5:**
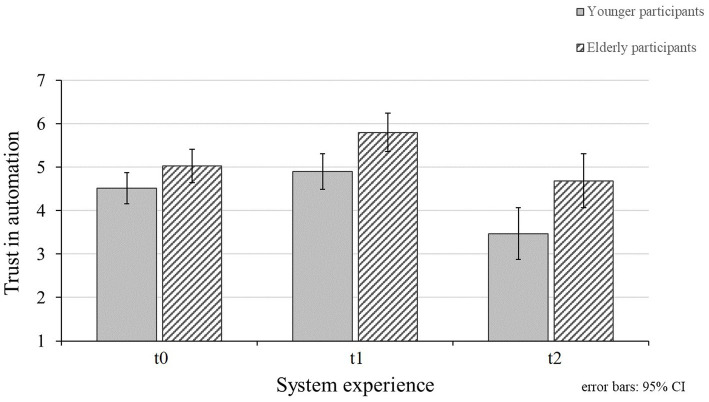
Mean values for younger and elderly participants’ trust in the eHMI for the points of measurement (t0 = initial measurement; t1 = valid system functions; and t2 = valid and invalid system functions). Higher values represent higher trust ratings.

The effect of the initial measurement (t0), after interacting with a valid system (t1), and after experiencing valid and invalid system functions (t2) on participants’ trust in the eHMI was examined (RQ1). Figure 5 displays the mean values and standard deviations for participants’ trust ratings for the different measurements and for both age groups. The conducted ANOVA revealed significant differences in trust ratings for the points of measurement (Table 3). Participants’ initial trust ratings of the eHMI are above the midpoint of the rating scale representing a rather moderate trust in the eHMI (*M_t0_* = 4.75; *SD_t0_* = 0.81). Data revealed an increase of trust after interacting with a reliable system (*M_t1_* = 5.32, *SD_t1_* = 0.99; Bonferroni-corrected pairwise comparison t0 and t1: *p* < 0.001), which supports H1a. Moreover, the ratings significantly decreased beyond the initial trust level after experiencing eHMI malfunctions (*M_t2_* = 4.04, *SD_t2_* = 1.39; Bonferroni-corrected pairwise comparisons t0 and t2: *p* = 0.004; t1 and t2: *p* < 0.001). Therefore, the data support H1b. In addition, a significant effect in trust ratings was found for the age groups (Table 3). In detail, elderly participants indicated significantly higher trust ratings toward the eHMI (*M_elderly_* = 5.17, *SD_elderly_* = 0.66) than younger participants did (*M_younger_* = 4.29, *SD_younger_* = 0.85). No significant interaction effect of trust ratings for the different points of measurement and participants’ age could be shown (Table 3).

### Acceptance of the eHMI

**Figure 6:**
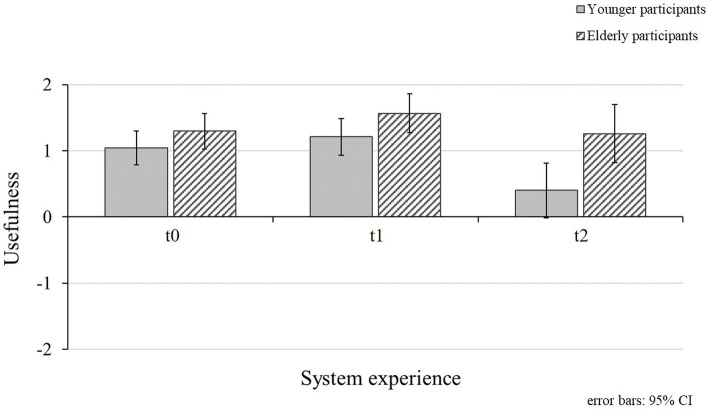
Mean values for younger and elderly participants’ usefulness ratings for the different points of measurement (t0 = initial measurement; t1 = valid system functions; and t2 = valid and invalid system functions). Higher values represent higher usefulness ratings.

**Figure 7:**
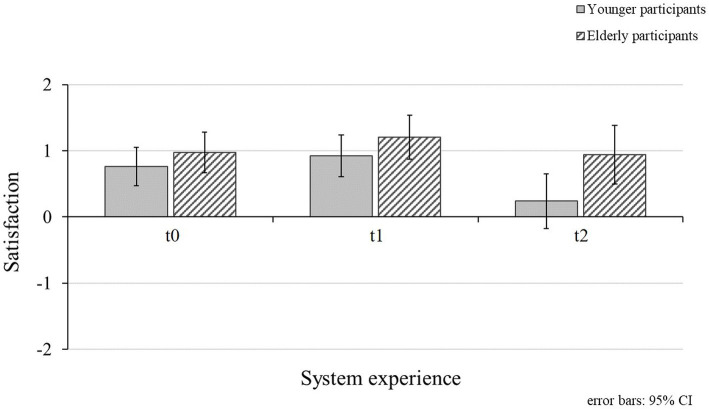
Mean values for younger and elderly participants’ satisfaction ratings for the different points of measurement (t0 = initial measurement; t1 = valid system functions; and t2 = valid and invalid system functions). Higher values represent higher satisfaction ratings.

Participants’ acceptance (i.e., comprising the subscales *usefulness* and *satisfaction*) of the eHMI (RQ2) was investigated during the initial measurement (t0), when interacting with the eHMI exclusively comprising valid system functions (t1) and after experiencing valid and invalid system functions (t2). Descriptive measures of participants’ acceptance ratings divided by age group are displayed in Figure 6 (subscale *usefulness*) and Figure 7 (subscale *satisfaction*).

#### Usefulness

For the eHMI *usefulness* ratings at the different points of measurements, the ANOVA uncovered a significant main effect (Table 3). Participants initially evaluated the investigated eHMI as rather useful (*M_t0_* = 1.16; *SD_t0_* = 0.55). Post-hoc comparisons (Bonferroni-corrected) showed that the *usefulness* ratings for the eHMI significantly increased when interacting with a valid system (*M_t1_* = 1.38; *SD_t1_* = 0.61) compared to the initial measurement (t0 and t1: *p* = 0.036; Bonferroni-corrected). After experiencing invalid system functions, participants’ *usefulness* ratings declined significantly in comparison with a valid system (*M_t2_* = 0.81; *SD_t2_* = 0.98; t1 and t2: *p* < 0.001; Bonferroni-corrected), which supports H2. However, there was no significant difference in participants’ usefulness ratings between the initial measurement and after experiencing system malfunctions (t0 and t2: *p* = 0.073). The investigated age groups evaluated the eHMI as significantly different regarding its *usefulness* (RQ5; Table 3). Specifically, elderly rated the eHMI as more useful (*M_elderly_* = 1.37, *SD_elderly_* = 0.60) than younger participants (*M_younger_* = 0.88, *SD_younger_* = 0.52). In addition, a significant interaction effect was obtained (Table 3). In this context, the stronger decline of younger participants’ usefulness ratings after experiencing eHMI malfunction compared to the elderly group should be highlighted (Figure 6).

#### Satisfaction

A significant main effect for participants’ *satisfaction* with the eHMI was shown for the different points of measurement (Table 3). Participants assessed the investigated eHMI as rather satisficing during the initial measurement (*M_t0_* = 0.86; *SD_t0_* = 0.62). Bonferroni-corrected pairwise comparisons revealed a significant decrease of the ratings after experiencing system malfunctions (*M_t2_* = 0.57; *SD_t2_* = 0.95) compared to valid system functions (*M_t1_* = 1.06; *SD_t1_* = 0.68; t1 and t2: *p* < 0.001; Bonferroni-corrected). Based on the results, H2 could be confirmed. There was no significant difference in ratings between the other points of measurement (t0 and t1: *p* = 0.086; t0 and t2: *p* = 0.131; Bonferroni-corrected). Participants’ age group did not appear to influence the *satisfaction* ratings of the investigated eHMI significantly (RQ5; Table 3). However, there was a significant interaction effect between participants’ age group and *satisfaction* ratings for the different measurements (Table 3). Similar to the effect obtained for usefulness, this result was mainly driven by the stronger decline of *satisfaction* scores of younger participants after experiencing invalid eHMI functions compared to the ratings by the elderly participants (Figure 7).

### Feeling of Safety During the Interaction

**Figure 8:**
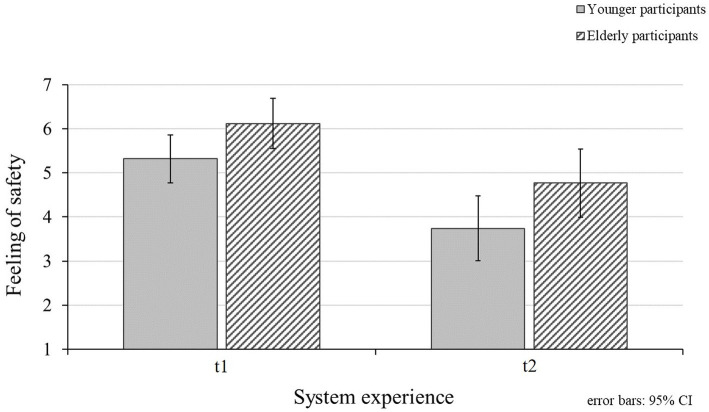
Mean values for younger and elderly participants’ feeling of safety ratings during the interaction for the different points of measurement (t1 = valid system functions; t2 = valid and invalid system functions). Higher values represent higher feeling of safety.

Besides assessing the eHMI, participants indicated their feeling of safety during the encounters with the vehicle (RQ3; Figure 8) after interacting with a valid system (t1) and after experiencing invalid eHMI functions (t2). During the interactions with valid system functions, participants indicated to feel rather safe (*M_t1_* = 5.69; *SD_t1_* = 1.22). However, feeling of safety declined significantly after interacting with an invalid system (*M_t2_* = 4.22; *SD_t2_* = 1.64; Table 3). Moreover, a significant difference between the age groups for feeling of safety during the encounter with the vehicle was revealed (RQ5; Table 3). In detail, elderly participants indicated a higher feeling of safety during the interactions (*M_elderly_* = 5.44, *SD_elderly_* = 1.01) compared to younger participants (*M_younger_* = 4.53, *SD_younger_* = 1.40). There was no significant interaction effect between participants’ feeling of safety ratings for the different points of measurements and the investigated age groups (Table 3).

### Vigilance Toward the eHMI

**Figure 9:**
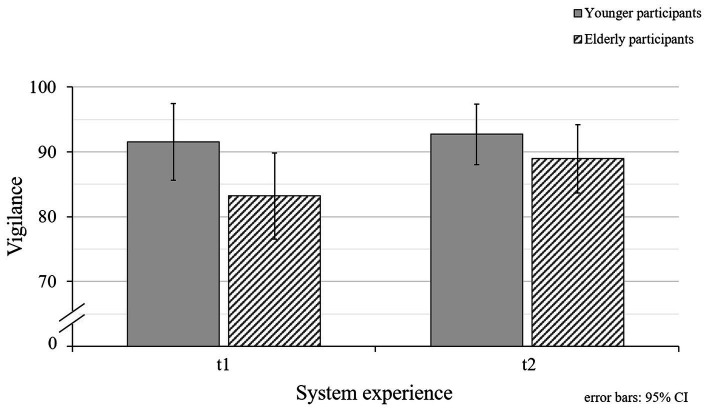
Mean values for younger and elderly participants’ vigilance toward the eHMI for the different points of measurement (t1 = valid system functions; t2 = valid and invalid system functions). Higher values represent higher indicated vigilance toward the system.

In addition, participants’ vigilance toward the eHMI (RQ4) was examined after experiencing valid eHMI functions (t1) and after interacting with an invalid system (t2) as an indicator for participants’ awareness of potential system malfunctions (RQ4). Generally, participants’ indicated to be rather observant regarding the eHMI signals (Figure 9). However, the ratings even increased significantly when experiencing eHMI malfunctions (*M_t2_* = 91.06, *SD_t2_* = 10.03) compared to valid system functions (*M_t1_* = 87.85, *SD_t1_* = 13.16; Table 3). The impact of eHMI malfunctions showed to be relevant for both age groups, since there was neither a significant main effect for participants’ age groups (RQ5; Table 3) nor an interaction effect between vigilance ratings for the different points of measurement and participants’ age groups found (Table 3).

## Discussion

The present study investigated the effects of eHMI malfunctions (i.e., mismatches between vehicle’s movements as implicit communication cues and explicit eHMI signals) on younger and elderly participants’ assessment of the system. Previous research reported that participants indicated higher feeling of safety (de Clercq et al., 2019) and trust (Faas et al., 2020) during interactions with AVs when eHMI signals were presented compared to interactions comprising exclusively implicit communication signals. Therefore, participants’ overtrust in case of eHMI malfunctions could display a potential safety issue in AVs (Tabone et al., 2021). Due to ambiguous findings regarding elderly users’ attitudes toward technology (Czaja et al., 2006; Schaefer et al., 2016), age-related differences of eHMI assessment and potential system malfunctions were investigated within the present study. Participants indicated their trust and acceptance of the eHMI, feeling of safety during the interaction and vigilance toward the eHMI across different points of measurement including valid and invalid system functions. Results showed that participants’ assessment of the eHMI increased with experience regarding trust and acceptance (i.e., usefulness ratings) compared to the initial measurement. Participants’ trust, acceptance, and feeling of safety declined significantly after experiencing eHMI malfunctions, whereas participants’ vigilance toward the eHMI increased after the experienced malfunctions. Moreover, elderly participants indicated significantly higher trust, acceptance (i.e., usefulness ratings), and feeling of safety ratings across all conditions compared to younger participants.

Generally, participants assessed eHMI signals as useful means of communication in AVs. This is reflected in rather high levels of trust and acceptance ratings during the initial measurement. The results are in line with previous findings (Rouchitsas and Alm, 2019). As expected, participants’ trust in the system increased after interacting with a valid system (H1a), since system experience can be described as an influencing factor of users’ *learned trust* according to Hoff and Bashir (2015). Despite a rather short period of achieving system experience in the current study, a similar development was also shown in previous research that included a longer period of three weeks to gain system experience with the investigated eHMI (Faas et al., 2020). However, participants’ trust and acceptance ratings of the system declined significantly when experiencing eHMI malfunctions as an additional component of *learned trust* (Hoff and Bashir, 2015). In line with the assumptions and previous studies considering eHMI malfunctions in crossing scenarios (Kaleefathullah et al., 2020; Faas et al., 2021), participants indicated lower trust ratings when experiencing invalid system functions (H1b). The current study applied a shared space scenario that comprised lower speed levels of the interaction vehicle. Therefore, implicit communication cues, such as the vehicle’s deceleration, might be more difficult to recognize due to lower encountering speeds and thus lower speed differences during deceleration maneuvers. However, despite the lower speed levels, participants seem to be sensitive regarding mismatches of implicit communication cues and eHMI signals. This awareness might potentially be necessary in shared space settings due to ambiguous encounters and a diversity of traffic participants that need to interact (Hamilton-Baillie, 2008).

As expected, participants’ acceptance of the eHMI as means of communication in AVs also declined significantly after experiencing invalid system functions that were not announced beforehand compared to exclusively valid system functions (H2). The investigated eHMI malfunctions might be comparable to omitted system failures of driving assistance systems as investigated by Beggiato and Krems (2013). The authors reported a decline of users’ acceptance when experiencing omitted system failures (Beggiato and Krems, 2013), as also shown in the current study with eHMI malfunctions. On the other hand, participants’ acceptance ratings remained rather moderate despite experiencing multiple eHMI malfunctions in the current study.

In addition, eHMI malfunctions also impaired participants’ feeling of safety during the interaction with the vehicle (RQ3). The results are in line with findings by Holländer et al. (2019) and developed similar to participants’ trust and acceptance ratings of the eHMI. Moreover, the participants indicated rather high vigilance ratings toward the eHMI, even when experiencing exclusively valid system functions. Moreover, the vigilance ratings increased after experiencing eHMI malfunctions (RQ4). This might be constituted in reduced trust in the eHMI due to the experienced malfunctions (Lee and See, 2004). The participants seem to be aware of additional monitoring requirements resulting in increased vigilance ratings in case of eHMI malfunctions to ensure traffic safety (Warm et al., 2008).

Regarding age effects (RQ5), an overall impact in terms of generally higher trust and perceived usefulness ratings, as one aspect of users’ acceptance, in the eHMI was found for elderly compared to younger participants. Moreover, elderly participants indicated a higher feeling of safety during the interaction with the vehicle. The results are in line with previous studies that reported higher trust ratings (Schaefer et al., 2016) and a more positive attitude toward automated systems of elderly users compared to younger users (Hartwich et al., 2019; Hensch et al., 2019b). However, within the current study, there were no differences in satisfaction ratings, as another factor of acceptance, between the investigated age groups. The result might be related to a general low intuitiveness of the applied eHMI signals that required an acquisition of the signals’ meanings (Hensch et al., 2019a). Considering repeated malfunctions of the eHMI, elderly participants indicated a tendency of overreliance in the eHMI. In particular, elderly participants still indicated higher trust and acceptance ratings (i.e., usefulness ratings) than younger participants when evaluating the eHMI after experiencing malfunctions. In addition, elderly users also indicated higher feeling of safety during the interaction with the vehicle when experiencing an invalid eHMI than younger users did. Despite experiencing repeated malfunctions of the eHMI, elderly participants adjusted their acceptance assessment of the system less when experiencing malfunctions, which was also shown for elderly users’ trust adjustment in previous research considering an automated decision aid (Ho et al., 2005). One explanation might be given by declines in working memory capacity of elderly (Salthouse, 1992). For the system assessment that was conducted block wise after 18 trails respectively, information about the frequency of malfunctions needed to be integrated in a mental representation of the system and recalled from working memory. Moreover, elderly may have difficulty in interpreting stochastic information, such as the probability of valid system functions and system malfunctions. Considering these aspects, the block wise system assessment might have led to a more positive assessment of the system by elderly participants (i.e., overestimating valid system functions in the overall mental representation of the system, since more trials displayed valid eHMI functions; Ho et al., 2005). Prospective studies should therefore collect participants’ assessment of the system in case of malfunctions in shorter time intervals (e.g., after each single trial) to prevent from distortion of the system assessment. It should be noted that participants of the current study did not perform ability checks (e.g., sensory and cognitive ability checks) that could support the given explanations of the current findings. However, the results are worrisome, since elderly pedestrians might be particularly imperiled by eHMI malfunctions, including possible safety issues, that are constituted in longer response and execution times to conduct actions in traffic scenarios (Stelmach and Nahom, 1992). Therefore, further studies are necessary to gain more information about the rationales of the obtained effects.

For ethical and safety reasons and to standardize the data collection process, the current study was conducted as a laboratory study with a therefore rather limited external validity. Moreover, the participants’ task to indicate their hypothetical crossing decisions by pressing a button to prevent from fatigue might have been rather artificial. It should be also mentioned that the current study neither conducted manipulation checks that controlled for participants’ adequate responses during the interaction with the investigated eHMI signals nor collected additional explanations for participants’ decisions to cross or not to cross. Therefore, the collection of additional behavioral measures, corresponding assessments, and explanations would be of interest in further studies. Moreover, the current study investigated two types of eHMI malfunctions that differed in the resulting criticality for pedestrians’ safety. In particular, the investigated malfunction F IV potentially impaired traffic safety, since the trajectories of the vehicle and the pedestrian hypothetically overlapped. Whereas malfunction F III did not directly impair traffic safety, since the participants’ instructed intention to cross the parking space was not compromised by the vehicle’s driving behavior (i.e., movement straight ahead the parking lot) or the displayed eHMI signal (i.e., crossing mode). Thus, the revealed declines in participants’ trust, acceptance, and feeling of safety ratings might be mainly driven by the examined safety critical malfunctions (i.e., F IV). However, even the experience of not directly safety relevant eHMI malfunctions might have affected the assessment and interaction with eHMIs to some extent, for instance in terms of a general acceptance and feeling of safety, since the participants experienced an unreliable system (Lee and Moray, 1992). When investigating the effects of malfunctions on participants’ eHMI assessment in further studies, the effect of safety critical malfunctions and non-critical malfunctions should be considered in a between-subjects design. In addition, the development of users’ system assessment over additional points of measurement, such as trust recovery, should prospectively be considered.

The findings of the current study showed that participants seem to be generally sensitive regarding eHMI malfunctions. Participants adjusted their assessment of the system due to the experienced malfunctions of the system. Since the vehicle’s driving behavior also represented a source of information in form of a dynamic HMI (Bengler et al., 2020), the results imply that participants considered the vehicle’s motion behavior as implicit communication cues in addition to the provided eHMI signals during the encounters with the vehicle. When applied as means of communication in AVs, eHMI signals are required to be in line with vehicle’s movements as implicit communication signals to benefit of the additional signals that could enhance traffic safety and support the interaction with surrounding traffic participants (Tabone et al., 2021). Moreover, the additional explicit signals could improve pedestrians’ situational awareness by supporting the predictability of prospective driving maneuvers conducted by the encountering AV (Endsley, 1995; Habibovic et al., 2018). To support traffic safety, an appropriate level of trust in eHMI signals, preventing for distrust and also overtrust, needs to be calibrated even system malfunctions are rare events (Lee and See, 2004). An appropriate system usage could be supported by preliminary information about the signals’ meanings. For instance, providing detailed information about the specific meaning of the applied eHMI signals can facilitate surrounding traffic participants to detect system malfunctions. Additional information, provided by eHMI signals, might also support the system’s transparency, which in turn could support traffic safety and the users’ acceptance of AVs (Faas et al., 2021).

## Conclusion

EHMIs offer the potential to support the interaction between AVs and pedestrians (Habibovic et al. 2018). However, potential eHMI malfunctions cannot be excluded in AVs (Holländer et al., 2019). With regard to traffic safety, pedestrians need to be aware of potential failures and are required to react appropriately by considering the vehicles’ implicit driving cues as a source of information in case of eHMI malfunctions. The findings of the current study imply that participants considered the vehicle’s movements as implicit communication cues in addition to the provided eHMI signals in case of malfunctions, which is reflected in an adjusted assessment of the eHMI system. Thus, to support traffic safety and smooth interactions with surrounding traffic participants, eHMI signals are required to be in line with the vehicle’s movements as implicit communication signals when applied as means of communication in AVs (Tabone et al. 2021). Moreover, the results underline the importance of calibrating an appropriate level of trust and expectations in eHMI signals among traffic participants. Thereby, the requirements of different user groups, such as elderly pedestrians, should be specifically considered. In order to develop an adequate understanding of the system, preliminary information about eHMI signals need to be provided if the systems are applied in AVs (Faas et al., 2021).

## Data Availability Statement

The datasets presented in this article are not readily available because of the involvement of project partners and required confidentiality of data within the project. Requests to access the datasets should be directed to A-CH, ann-christin.hensch@psychologie.tu-chemnitz.de.

## Ethics Statement

Ethical review and approval was not required for the study on human participants in accordance with the local legislation and institutional requirements. The patients/participants provided their written informed consent to participate in this study.

## Author Contributions

A-CH: conceptualization, methodology, formal analysis, investigation, writing—original draft, and visualization. IK: conceptualization, methodology, formal analysis, investigation, writing—original draft, writing—review and editing, visualization, and supervision. MB: conceptualization, methodology, software, investigation, writing—original draft, writing—review and editing, and supervision. JK: investigation, writing—review and editing, supervision, and project administration. All authors contributed to the article and approved the submitted version.

## Funding

The research was funded by the Bundesministerium für Verkehr und digitale Infrastruktur (BMVI, German Federal Ministry of Transport and Digital Infrastructure; grant no 16AVF2016A)—project “InMotion—Light-based communication between automated vehicles and other road users”; and by the Deutsche Forschungsgemeinschaft (DFG, German Research Foundation)—Project-ID 416228727—SFB 1410.

## Conflict of Interest

The authors declare that the research was conducted in the absence of any commercial or financial relationships that could be construed as a potential conflict of interest.

## Publisher’s Note

All claims expressed in this article are solely those of the authors and do not necessarily represent those of their affiliated organizations, or those of the publisher, the editors and the reviewers. Any product that may be evaluated in this article, or claim that may be made by its manufacturer, is not guaranteed or endorsed by the publisher.
